# Functional Properties of Rice Bran Proteins Extracted from Low-Heat-Treated Defatted Rice Bran

**DOI:** 10.3390/molecules27217212

**Published:** 2022-10-25

**Authors:** Seong-Jun Cho, Sang-Deok Lee, Sung-Wook Han

**Affiliations:** 1 Department of Food Science and Biotechnology, Kangwon National University, Kangwondaehak-gil, Chuncheon 24341, Gangwon-do, Korea; 2 Division of Forest Science, Kangwon National University, Kangwondaehak-gil, Chuncheon 24341, Gangwon-do, Korea; 3 CJ Cheiljedang Blossom Park, Gwanggyo-ro 42, Suwon-si 16945, Gyeonggi-do, Korea

**Keywords:** rice bran, protein isolates, low-heat treatment, functional properties

## Abstract

Rice bran is rich in proteins with high nutritional values. However, current protein extraction methods from rice bran are greatly limited by their low yield. Therefore, in this study, we aimed to develop a feasible method to extract rice bran protein (RBP) of high purity and quality. We prepared RBP using low-heat-treated defatted rice bran (LDRB) and analyzed its functional properties. The protein solubility of LDRB increased from 25.4% to 56% upon increasing the pH level and was more than double that of heat-stabilized defatted rice bran. RBP prepared from LDRB had good functional properties, comparable to those of soy proteins. The emulsifying capacities of RBP were 424 ± 14 mL/g at pH 4 and 530 ± 21 mL/g at pH 7.0. Under acidic conditions, RBP showed a better emulsifying capacity than soy proteins (262 ± 1 mL/g at pH 4). RPB showed water-binding and oil-absorption capacities of 270 ± 35 g/100 g and 268 ± 30 g/100 g, respectively. Moreover, RBP showed better foaming capacity (610% vs. 590%) and foam stability (83% vs. 4%) than soy proteins; however, it lacked gelling properties. This study demonstrated that RBP is a potential new protein source in the food industry.

## 1. Introduction

Rice bran, a by-product of rice processing, contains various nutrients including proteins, fats, carbohydrates, and micronutrients such as vitamins, minerals, antioxidants, and phytosterols [1,2]. Rice bran is an attractive source of protein because it is protein-rich (10–15%), plentiful, and inexpensive [3]. Rice bran protein (RBP) and its hydrolysate have high nutritional value and exhibit hypoallergenic, anticancer, and cholesterol-lowering effects as well as antioxidant properties [4,5,6,7]. However, protein extraction from rice bran is considerably challenging.

The proteins in rice bran are strongly bonded by disulfide linkages, forming high molecular complexes, which decrease their solubility in water. Rice bran contains a large content of lipids (up to 20%), which are easily degraded by lipase, leading to rancidity. Heat treatment is generally used to inactivate lipase; however, this treatment can cause protein denaturation and enhance the linkages between proteins and other components, leading to reduced protein extractability and decreased protein purity [8,9]. The alkaline extraction method is the most common and feasible procedure for protein extraction from rice bran; however, this method shows low protein extractability and purity from heat-stabilized defatted rice bran (HDRB) [10]. Several methods, including high pressure, sonication, hydrothermal cooking, and enzyme treatments, have been used to improve the protein extractability of rice bran and the functional properties of RBP [9,11,12,13]. Still, little attention has been paid to the use of low-heat-treated defatted rice bran (LDRB), which is prepared by solvent defatting of freshly milled rice bran without heat treatment, to prepare RBP.

Herein, we developed a feasible method to produce high purity and quality RBP using LDRB and characterized the functional properties of RBP, including protein solubility, emulsifying capacity, and water- and oil-binding capacity. This study provides fundamental information on the possible applications of RBP in food.

## 2. Results and Discussion

### 2.1. Protein Content and Solubility

**Figure 1:**
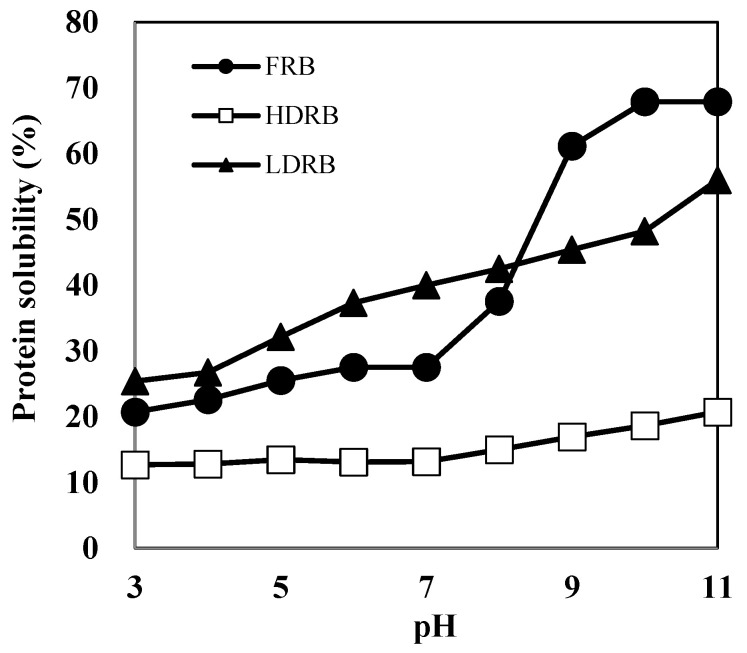
Protein solubility of three types of rice bran samples: FRB, full-fat rice bran; HDRB, heat-stabilized defatted rice bran; LDRB, low-heat treated defatted rice bran.

The protein content of RBP prepared from LDRB was 81.7%. Chandi and Sogi [10] have reported that the protein content of RBPs prepared by alkaline extraction is ~52.5–58.9%. Because heat treatment enhances the linkage between proteins and other components in rice bran, RBPs from heat-stabilized defatted rice bran (HDRB) contain several other components and show low protein content. Furthermore, protein extraction using HDRB is more challenging than that using LDRB because the protein solubility of HDRB is lower than that of LDRB (Figure 1).

**Figure 2:**
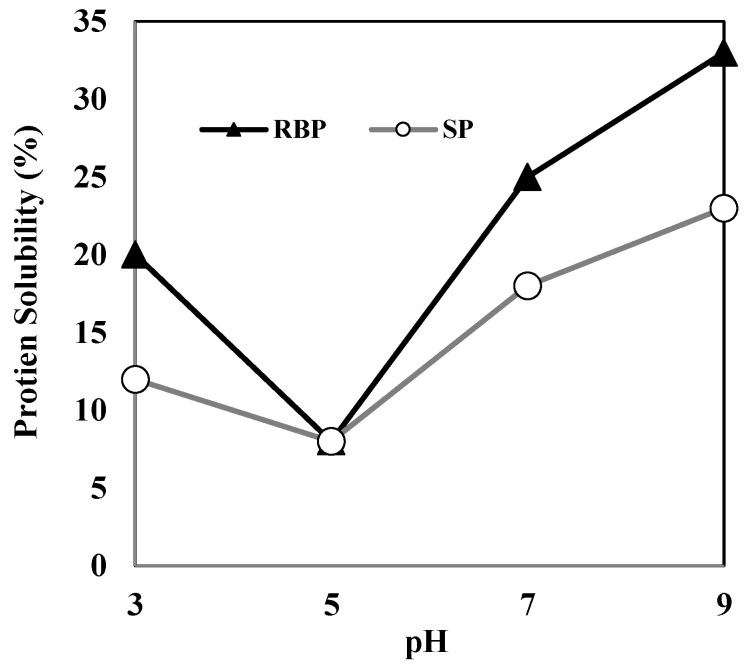
Protein solubility of RBP and SP at various pH conditions. RBP, rice bran protein; SP, soy protein.

Herein, the protein solubility of HDRB remained below 20% over the entire pH range (3.0–11.0), indicating that most of the HDRB proteins are denatured and insoluble. LDRB showed a considerably better protein solubility than HDRB; the solubility of LDRB increased two-fold, reaching a value of 43–48% at pH 8–10 and approximately 56% at pH 11. This indicates that mild processing conditions are necessary to render defatted bran a possible substrate for protein extraction. The high purity of RBP extracted from LDRB might be due to low protein denaturation and high protein solubility. The protein solubilities of RBP and soy protein (SP) are shown in Figure 2. The high solubility of protein facilitates its functional properties such as emulsifying, foaming, and gelation, which are not easily attainable in insoluble proteins [14]. The protein solubility of RBP was 20–33% at pH 3–9, which was higher than that of SP. This suggests that RBP may be better suited than SP for liquid food products.

### 2.2. Emulsifying Capacity

The emulsifying capacity (EC) of RBP was measured under acidic (pH 4) and neutral (pH 7) conditions and compared with that of SP and sodium caseinate (Table 1). SP is a good vegetable emulsifier under neutral conditions; however, the EC of SP significantly decreases from 845 to 262 mL/g under acidic conditions. In contrast, the EC of RBP is relatively stable under both acidic and neutral pH conditions, higher than that of SP and similar to that of sodium caseinate under acidic conditions. EC is mainly dependent on the interactions between proteins and lipids [15] and is related to protein structure. Therefore, these results show that RBP has a stable structure under acidic and neutral conditions and can be used as a vegetable emulsifier for acidic food.

### 2.3. Water- and Oil-Binding Capacities

**Figure 3:**
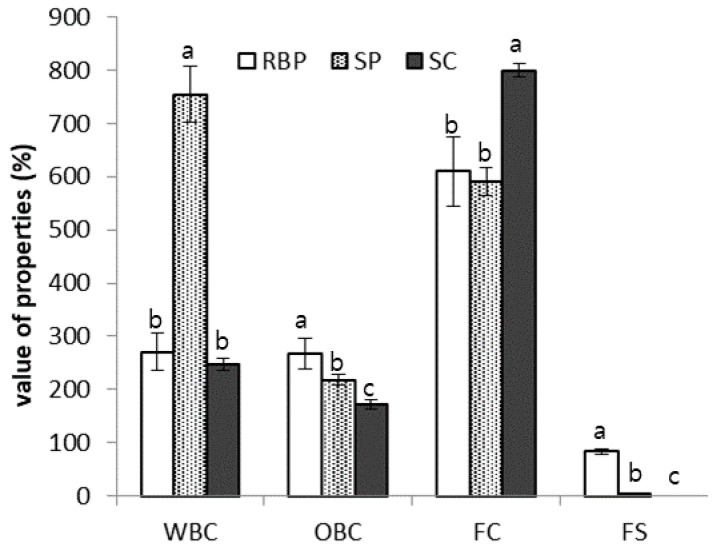
Water-binding capacity (WBC), oil-binding capacity (OBC), foaming capacity (FC), and foam stability (FS) of rice bran protein (RBP), soy protein (SP), and sodium caseinate (SC). Different letters indicate significant differences at *p* < 0.05.

The water-binding capacities (WBCs) and oil-binding capacities (OBCs) of various proteins, including RBP, are shown in Figure 3. The WBC count of RBP was 35.8% of that of SP and was similar to that of sodium caseinate. The protein solubility and WBC of a product are inversely proportional. As shown in Figure 2, the solubility of RBP was higher than that of SP; therefore, compared with SP, the low WBC of RBP can be explained by its high solubility.

The OBC of the RBP was significantly higher than that of SP and sodium caseinate (*p* < 0.05). Despite the high water solubility of RBP, its high OBC suggests the presence of an appreciable number of hydrophobic residues on the protein surface [16]. Considering that the distribution of hydrophobic and hydrophilic amino acids on the surface of the protein is a major factor in the WBCs and OBCs of proteins, the surface structure of rice protein and casein are similar; thus, rice protein may be used as a casein alternative.

### 2.4. Foaming Properties

The foaming activity and foam stability of RBP were measured and compared with those of SP, and the results are shown in Figure 1. The foaming activity of RBP was similar to that of SP; however, the foaming stability of RBP was considerably greater than that of SP, which was not stable over time.

Foaming stability is related to the stability of the film, which helps entrapped air bubbles remain intact. Because the lack of repulsive interactions of proteins improves the formation of a viscous film at the interface of the foam, protein foams are stabilized at the isoelectric point [17]. Therefore, this result indicates that RBP contains some protein fractions with an isoelectric point of approximately pH 7, improving the foam stability of RBP.

### 2.5. Gelling Properties

**Figure 4:**
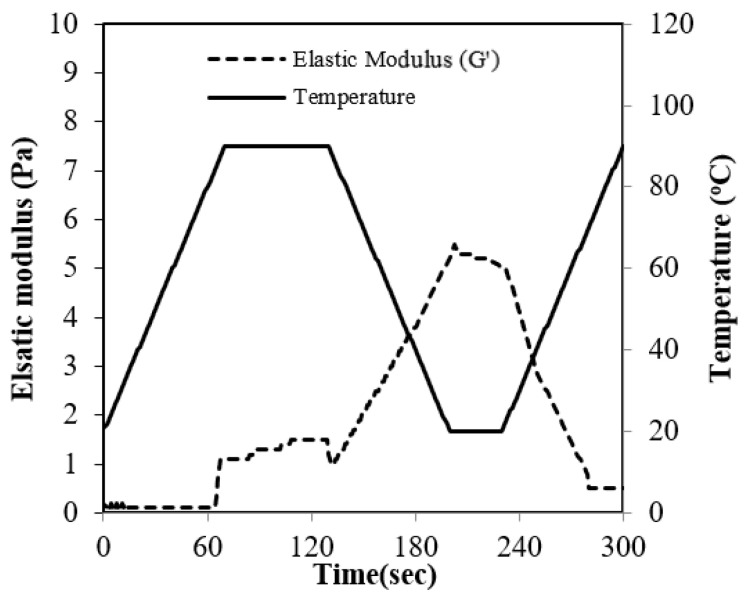
Elastic modulus (G′) of the rice bran protein solution during heat-induced gelation.

Under standard conditions, RBP exhibited almost no gelling behavior, as demonstrated in Figure 4. The resulting weak structure became evident when compared with the strong gelling property of SP, which showed an elastic modulus approximately 650 times higher (data not shown). During the heating period from 20 to 85 °C there was no increase in the elastic modulus, Because the RBP gel transition started from over 85 °C. During the cooling period, the elastic modulus had a steep increase due to the development of the gel structure during the formation of hydrogen bonds [18].

The low gelling properties of RPB suggest that the structure of RBP is highly stable because the gelation properties of the protein are negatively influenced by decreasing protein-unfolding characteristics.

## 3. Materials and Methods

### 3.1. Materials

Full-fat rice bran (*Oryza sativa* L.) and HDRB were obtained from Beidahwang Company (Harbin, China). Defatted soy flour (*Glycine max*) was obtained from Sigma–Aldrich (St. Louis, MO, USA). All other reagents were of analytical grade.

### 3.2. Protein Preparation

RBP was prepared from LDRB, and preliminary tests were conducted to optimize the defatting conditions to maximize the protein solubility of the defatted rice bran. LDRB was prepared by solvent extraction in a Soxhlet apparatus using hexane (rice bran to hexane at a ratio of 1:5, *w*/*v*; 50 °C), followed by air-drying in a fume hood. LDRB was thoroughly mixed with five-time distilled water. The mixture was adjusted to pH 9.0 with 1 N NaOH solution, stirred for 1 h at 30 °C, and centrifuged at 5000× *g* for 20 min. The supernatant was adjusted to pH 4.5 using 1 N HCl, maintained at 30 °C for 30 min for protein precipitation, and centrifuged at 5000× *g* for 20 min. The obtained solid residues were washed five times with distilled water and neutralized with 1 N NaOH. The neutralized protein solution was then dried using a spray dryer (KL-8, Seogang Engineering, Cheonan, Korea). Soy protein was prepared from commercial defatted soy flour using the same method as that used for RBP.

### 3.3. Protein Analysis

The protein content of the samples was determined by the Kjeldahl method [19] using an autoanalyzer (Kjeltec8200, Foss, Hillerod, Denmark) and Kjeldahl nitrogen was multiplied by a factor of 6.25 to obtain the total protein content.

### 3.4. Protein Solubility

The protein solubility of rice bran and RBP in water was determined using the method described by Jeong et al. [20] with some modifications. Distilled water (200 mL) was added to 0.2 g of the protein sample and mixed for 20 min with a magnetic stirrer. The pH of the mixture was adjusted to the desired level with 0.1 N HCl or 0.1 N NaOH, and the final volume of the mixture was adjusted to 250 mL. The mixture was then agitated for 5 min and centrifuged at 10,000× *g* for 30 min. The protein content in the supernatants was determined using the Kjeldahl method, and protein solubility was calculated as follows:

(1) $$\mathrm{Protein}\;\mathrm{solubility}\;\left(\%\right)=\frac{Protein\;content\;in\;the\;supernatant}{Total\;protein\;content}\times 100$$

### 3.5. Emulsifying Capacity (EC)

The EC of the rice protein was measured according to the method described by Sha et al. [21]. The protein solution (1% *w*/*w*) was stirred at a constant temperature (20 °C) in a 1 L laboratory reactor with a stirrer. Soybean oil was added dropwise to the protein solution and emulsified with a high-speed homogenizer (HG-15A, Daihan, Wonju, Korea) at 20,000 RPM continuously. The conductivity of the emulsion was continuously measured with a pH/conductivity multimeter (SevenDirect SD23, Mettler Toledo, Nanikon, Switzerland) and used as a parameter to determine its inversion point. The amount of oil added to the inversion point of the emulsion was used to calculate the emulsifying capacity (mL oil/g sample).

### 3.6. Water- and Oil-Binding Capacities

The WBC and OBC of the protein were measured according to the method described by Jeong et al. [20] with some modifications. One gram of each sample was dispersed in 10 mL of distilled water or soybean oil and placed in 50 mL centrifuge tubes. The mixture was dispersed using a vortex mixer to ensure thorough mixing. After a holding period of 30 min, the dispersions were centrifuged at 3000× *g* for 10 min. The supernatant was discarded and the pellet was weighed. WBCs and OBCs were expressed as grams of water or oil retained per 100 g of protein sample.

### 3.7. Foaming Properties

Foaming capacity (FC) and foam stability (FS) were determined using the method described by Cano-Median et al. [22] with some modifications. One gram of protein sample was added to 20 mL of distilled water (5% *w*/*v*) and adjusted to pH 7. The suspension was mixed vigorously for 2 min using a high-speed homogenizer (HG-15A) at 16,000 rpm. To measure the foam stability, the foams were immediately poured into a 500 mL mass cylinder, and the foam volume was measured after 60 min. 

The two foaming properties were determined using the following equations:

(2) $$\mathrm{FC}\;\left(\%\right)=\frac{V_{0}-\;V}{V}\times 100$$

$$\mathrm{FS}\;\left(\%\right)=\frac{V_{30}}{V_{0}}\times 100$$

where V is the volume of the solution before homogenization, *V*_0_ is the volume of the solution after homogenization, and *V*_30_ is the volume of the solution 30 min after homogenization.

### 3.8. Gelling Properties

The elastic modulus (G′) was determined by oscillating, non-destructive rheological measurements with a Bohlin CVO 1000 Rhenometer (Malvern Instruments Ltd., Worcestershire, UK) using a coaxial cylinder. Measurements were performed at a frequency of 0.1 Hz and 1% strain. Gel formation was induced by increasing the temperature from 20 to 90 °C at a constant heating rate of 1 K/min. The sample was maintained at 90 °C for 60 min and subsequently cooled to 20 °C at a cooling rate of 1 K/min. Reversible thermal properties were determined after maintaining the sample for 30 min at 20 °C and subsequent heating to 90 °C at a heating rate of 1 K/min [23].

### 3.9. Statistical Analysis

All results were subjected to a one-way analysis of variance (ANOVA) using SigmaStat (v.3.5. Systat Software Inc., Palo Alto, CA, USA). Statistical significance was set as *p* < 0.05, according to Duncan’s new multiple range test.

## 4. Conclusions

Herein, it was shown that LDRB is an effective raw material for the preparation of RBP. Because LDRB shows better protein solubility than HDRB, proteins in LDRB are easily extracted and isolated. Therefore, RBP prepared from LDRB had a higher protein content than that prepared from HDRB. There are many enzymatical and mechanical approaches to obtain high-protein content RBP including enzyme treatment, ultra-sonification, and electrolyzed water treatment; accordingly, LDRB is an eco-friendly and cost-effective way to produce high-protein content RBP. The functional properties of RBP from LDRB were different to that from soy protein and casein; notably, the emulsifying and foaming properties of RBP were superior to those of soy protein and casein. RBP has a low gel-forming ability compared with its high solubility. These results suggest that RBP can be applied to several types of emulsified foods such as mayonnaise, desserts, bakery products, and drinks. Recently, there has been an increasing need for new plant-based proteins and this study offers possibilities for RBP as a new protein source in the food industry.

Further study is required to understand the molecular structure of the unique properties of RBP.

## Figures and Tables

**Table 1:** The emulsifying capacity of rice bran protein, soy protein, and sodium caseinate at pH 4 and 7.

	Emulsifying Capacity (mL/g)		
	Rice Bran Protein	Soy Protein	Sodium Caseinate
pH 4	424 ± 14.2 a	262 ± 11.3 b	425 ± 13.1 a
pH 7	530 ± 21.4 c	845 ± 41.8 a	745 ± 21.2 b

Different letters indicate significant differences, *p* < 0.05.
